# Determinants of fertility intentions in a low-fertility region of Central Europe: a cross-sectional survey of 1,092 respondents in the Greater Poland Voivodeship

**DOI:** 10.3389/fpubh.2026.1872501

**Published:** 2026-07-07

**Authors:** Andrzej Ciborek, Zuzanna Chęcińska-Maciejewska, Agnieszka Nowak, Hanna Krauss

**Affiliations:** 1 Department of Interprofessional Education, Faculty of Medicine and Health Sciences, Kalisz University of President Stanisław Wojciechowski, Kalisz, Poland; 2 Department of Food and Nutrition, Kalisz University of President Stanisław Wojciechowski, Kalisz, Poland; 3 Faculty of Medicine and Health Sciences, Kalisz University of President Stanisław Wojciechowski, Kalisz, Poland; 4 Department of Basic Sciences, Institute of Preventive Research, Kalisz University of President Stanisław Wojciechowski, Kalisz, Poland

**Keywords:** Central Europe, childbearing intentions, factor analysis, family policy, Greater Poland Voivodeship, ordinal regression, Poland, reproductive plans

## Abstract

Persistent below-replacement fertility and accelerated population ageing now drive much of the public health agenda in Europe, with measurable consequences for perinatal services and long-term care demand. Aggregate-level data document these trends well; individual-level determinants of fertility intentions in Central European low-fertility settings remain less well characterised. We carried out a cross-sectional online survey in the Greater Poland Voivodeship of Poland between March 2023 and March 2024, recruiting through mixed online channels. The instrument covered demographic, socioeconomic, residential, normative and behavioural domains, including a 10-item Likert scale on perceived barriers to childbearing. The primary outcome was the desired number of children “regardless of existing barriers.” Associations were assessed using Spearman correlation, Kruskal–Wallis and Mann–Whitney tests, and proportional-odds ordinal logistic regression; perceived barriers were summarised by exploratory factor analysis. The sample comprised 1,092 respondents aged 18 to over 40 years, recruited across the rural–urban gradient. The mean desired number of children was 1.84 (SD 1.09); 14.8% wanted no children, while 22.9% wanted three or more. In adjusted ordinal logistic regression, religion influencing family decisions (OR = 2.39; 95% CI 1.75–3.27; *p* < 0.001), receipt of the 500+ child benefit (OR = 1.58; 95% CI 1.26–1.99; *p* < 0.001) and age (OR = 1.03 per year; 95% CI 1.02–1.04; *p* < 0.001) were positively associated with higher reproductive intentions, while urbanisation level was negatively associated (OR = 0.92; 95% CI 0.86–0.99; *p* = 0.023). Subjective financial situation, educational attainment and perceived influence of the Ukraine war were not significant after adjustment. Factor analysis of the 10 barrier items (KMO = 0.86; Bartlett *p* < 0.001) identified two dimensions: structural barriers (*α* = 0.84; 46.4% of variance), encompassing material situation, housing, partner availability and career ambitions; and psychosocial-normative barriers (*α* = 0.80; 13.1% of variance), encompassing fear of parenthood, perceived lack of support and reluctance to change one’s current life. Fertility intentions in this Central European setting are shaped by a heterogeneous mix of structural, normative and life-course factors that cannot be reduced to material conditions alone, supporting a multi-dimensional approach to public health and family policy.

## 1 Introduction

Below-replacement fertility across most of Europe is no longer an emerging trend. It is a structural feature of the continent’s demography (1, 2), with consequences that reach well beyond the field of demographic statistics. Falling numbers of live births reshape the age structure of the population, alter the demand for perinatal, paediatric and long-term care, and place pressure on the financial sustainability of social insurance systems that depend on the ratio between contributing and dependent populations (3, 4). Within Europe, Poland is currently among the countries with the lowest period fertility; the national total fertility rate fell to a post-war minimum of 1.099 in 2024 (5).

Aggregate-level analyses of these trends have grown in number and depth. Companion regional work in the Greater Poland Voivodeship has shown that live births fell by 31.9% between the 2017 peak and 2023, and that the parallel improvement in macroeconomic conditions did not coincide with any recovery in fertility (6). Such ecological evidence leaves a fundamental question open. What do individuals—the women and men whose decisions ultimately produce these aggregates—say about their own reproductive intentions? Which structural, material, normative and life-course factors do they actually perceive as relevant to those decisions?

Work on fertility intentions in Central and Eastern Europe has grown rapidly over the past decade, especially after the COVID-19 pandemic and against the backdrop of broader normative shifts in the region. Marcinkowska et al. (7) documented a measurable decline in reproductive desires among Polish women during the pandemic and linked part of that decline to perceived restrictions on reproductive rights. Tymicki et al. (8) traced the short-term impact of the pandemic on age-specific fertility rates in Poland; the strongest effects appeared among women in their late twenties and early thirties. At the European level, Vignoli et al. (9) advanced a narrative framework that ties perceived uncertainty about the future—economic, political, normative—to fertility postponement, and Comolli et al. (10) demonstrated that the consequences of macro-level shocks on fertility extend well beyond the duration of the shock itself.

Several theoretical perspectives help to interpret individual-level fertility intentions. Esping-Andersen and Billari (11) argued that fertility recovery in low-fertility settings depends primarily on the diffusion of gender-egalitarian norms, not on short-term economic improvement. Beaujouan (12) showed that the postponement and resurgence of late parenthood across low-fertility countries reflects a complex interaction of cohort, period and tempo effects. Bergsvik et al. (13), in a systematic review of (quasi-)experimental evidence, concluded that childcare expansions appear to increase completed fertility, while increased cash transfers have only temporary effects. The latter conclusion has direct relevance for the Polish 500+ child benefit, introduced in 2016 and substantially expanded thereafter.

The motivation for this study is threefold. First, individual-level evidence on fertility intentions in Central European low-fertility regions is sparse, and most published Polish work has used national samples rather than the within-region heterogeneity of large voivodeships such as Greater Poland (14). Second, the relationship between subjective economic conditions, transfer programmes such as 500+ and self-reported reproductive intentions has not been formally tested in adjusted multivariable models in this setting. Third, although extensive lists of perceived barriers to childbearing have been reported in the literature, the latent structure underneath those barriers—whether they cluster into distinct dimensions amenable to different policy responses—remains underexplored.

Our aim was therefore to characterise fertility intentions and their structural, normative and life-course determinants in a regional sample of women and men in Greater Poland, and to identify the latent dimensions underlying perceived barriers to childbearing. We hypothesised, on the basis of the literature reviewed above, that age, religion and place of residence would be associated with reproductive intentions; that subjective financial situation would not be a strong adjusted predictor; and that perceived barriers would be reducible to a small number of interpretable dimensions reflecting the distinction between material-structural and psychosocial-normative concerns.

## 2 Materials and methods

### 2.1 Study design and setting

We conducted a cross-sectional online survey in the Greater Poland Voivodeship of Poland between March 2023 and March 2024. The voivodeship covers approximately 29,826 km^2^ in west-central Poland and is home to roughly 3.5 million inhabitants distributed across 35 counties — from the Poznań metropolitan agglomeration to peripheral rural counties with markedly different demographic trajectories. The setting offers substantial within-region heterogeneity in the rural–urban gradient, educational composition and economic conditions, all of which are relevant to fertility intentions.

### 2.2 Recruitment and instrument

Recruitment proceeded through mixed online channels: social-media dissemination, university and institutional mailing lists, and snowball referral by initial respondents. The questionnaire was administered electronically using the Microsoft Forms platform and was completed anonymously. Eligibility criteria were residence in the Greater Poland Voivodeship, age 18 years or older, and consent to participate. The instrument had been piloted earlier by the same research group in a separate study of 419 respondents (data not reported here), which established the comprehensibility and internal consistency of the items.

The questionnaire comprised 44 items distributed across five domains: (i) socio-demographic characteristics (age category, sex, educational attainment, parental education, place of residence, family structure, family of origin); (ii) economic and housing situation (subjective financial situation, household income per person, housing situation, household density, receipt of social transfers); (iii) reproductive history and intentions (number of past pregnancies and births, contraceptive use, desired number of children, preferred family form, planning of next pregnancy); (iv) perceived barriers to childbearing, captured by a 10-item Likert instrument on a 0–5 scale ranging from “no influence” to “strongest influence,” covering material, partner-related, residential, occupational, normative and psychosocial domains; and (v) normative and contextual factors (influence of religion on family decisions, perceived influence of the war in Ukraine on reproductive plans, expectations regarding future life, attitudes towards family size).

### 2.3 Variables and operational definitions

The primary outcome was the desired number of children, captured by the question “Regardless of existing barriers, how many children would you like to have in your life?” with response categories “none,” “1,” “2,” “3,” “4” and “5 or more.” For the analysis this was treated as an ordinal variable with values 0–5.

Predictor variables of primary interest were defined as follows. Age was self-reported in five categories (18–20, 21–25, 26–30, 31–40, over 40 years) and treated as ordinal; for the regression model, midpoint values (19, 23, 28, 35, 45) were used as a continuous proxy. Educational attainment was captured in five categories (primary, vocational, currently studying, secondary, tertiary) and recoded into a four-point ordinal scale, with “currently studying” placed alongside “secondary” given the typical educational trajectory of respondents in this category. Place of residence was recorded across five categories (rural; town up to 20,000 inhabitants; 20,001–50,000; 50,001–150,000; over 150,000) and treated as ordinal urbanisation. Subjective financial situation was captured on a five-point scale (very bad, rather bad, average, good, very good). Religion was operationalised as a binary indicator of whether religion influences family decisions (yes versus no/unsure). Receipt of the Polish 500+ child benefit was recorded as a binary indicator. Perceived influence of the Ukraine war on reproductive decisions was a binary item.

The 10-item barrier instrument captured perceived influence of: (1) lack of a suitable partner; (2) being in an informal relationship; (3) partner’s reluctance to have children; (4) material situation; (5) career ambitions; (6) lack of suitable housing; (7) fear of parenthood; (8) lack of family support; (9) lack of state support; (10) reluctance to change one’s current comfortable life. Each item was rated on a Likert scale from 0 (no influence) to 5 (strongest influence).

### 2.4 Statistical analysis

Descriptive statistics were computed for all variables. For continuous and ordinal variables, means with standard deviations and medians were reported; for categorical variables, frequencies and percentages were reported.

Bivariate associations between predictor variables and the desired number of children were assessed using Spearman rank correlation for ordinal predictors, the Kruskal–Wallis test for unordered categorical predictors with three or more groups, and the Mann–Whitney U test for binary predictors.

The adjusted association between candidate predictors and the desired number of children was estimated using a proportional-odds ordinal logistic regression with the desired number of children (0–5) as the outcome and the following predictors: age (continuous proxy), educational attainment (ordinal), urbanisation level (ordinal), religion influencing family decisions (binary), subjective financial situation (ordinal), perceived influence of the Ukraine war (binary) and receipt of the 500 + child benefit (binary). Odds ratios are reported with 95% confidence intervals and two-sided *p*-values.

Latent dimensions underlying the 10-item barrier instrument were examined by exploratory factor analysis. Sampling adequacy was assessed using the Kaiser–Meyer–Olkin (KMO) statistic and Bartlett’s test of sphericity. The number of factors to retain was determined by the Kaiser criterion (eigenvalue greater than 1) and inspection of the scree plot. Factor extraction was performed by principal-component analysis, followed by varimax rotation. Internal consistency of the resulting factor-based scales was assessed by Cronbach’s alpha.

All analyses were performed in Python 3 using the pandas, scipy.stats, statsmodels and scikit-learn libraries. Statistical significance was defined as a two-sided *p*-value below 0.05. Reporting followed the STROBE statement for cross-sectional studies; the completed checklist is provided as Supplementary File S1.

### 2.5 Ethical considerations

The study was based on an anonymous online survey that did not collect identifiable personal information, did not involve any medical intervention, biological sampling or sensitive health data, and posed no foreseeable risk of harm to participants beyond what they would encounter in everyday life. Under Polish law and the regulations governing biomedical research in Poland (Act of 5 December 1996 on the Professions of Physician and Dentist, Art. 21–29, as amended), formal review by an institutional bioethics committee is required for medical experiments involving human participants and for research involving identifiable health data. Anonymous opinion surveys that do not collect sensitive personal data fall outside this scope and do not require formal bioethics committee approval. Participation was voluntary; respondents were informed of the purpose of the study and of their right to discontinue at any time, and informed consent was obtained electronically before respondents could access the questionnaire. No incentives were offered. The study was conducted in accordance with the principles of the Declaration of Helsinki and with applicable national regulations on the protection of personal data (GDPR, Regulation (EU) 2016/679).

## 3 Results

### 3.1 Sample characteristics

The total sample comprised 1,092 respondents (Table 1). The age distribution was skewed towards younger respondents, reflecting the recruitment channels: 32.9% were aged 18–20 years, 19.2% were aged 21–25, 11.5% were aged 26–30, 18.3% were aged 31–40, and 18.0% were over 40 years. With respect to educational attainment, 34.3% of respondents had completed tertiary education, 32.1% had completed secondary education, 26.0% reported being currently in education, 3.8% had completed vocational education, and 3.7% had completed only primary education. Place of residence was distributed across the rural–urban gradient: 32.8% lived in rural areas, 26.1% in cities with more than 150,000 inhabitants, and the remaining 41.1% in smaller towns and cities.

**Table 1:** Sociodemographic and economic characteristics of the sample (*N* = 1,092).

Variable	Category	*n*	%
Age (years)	18–20	359	32.9%
	21–25	210	19.2%
	26–30	126	11.5%
	31–40	200	18.3%
	Over 40	197	18.0%
Educational attainment	Tertiary	375	34.3%
	Secondary	351	32.1%
	Currently studying	284	26.0%
	Vocational	42	3.8%
	Primary	40	3.7%
Place of residence	Rural area	358	32.8%
	Town up to 20,000	159	14.6%
	Town 20,001–50,000	147	13.5%
	Town 50,001–150,000	143	13.1%
	City over 150,000	285	26.1%
Subjective financial situation	Very good	157	14.4%
	Good	511	46.8%
	Average	378	34.6%
	Rather bad	36	3.3%
	Bad	10	0.9%
Household income per person (PLN/month)	Up to 1,000	113	10.3%
	1,001–2,000	295	27.0%
	2,001–5,000	529	48.4%
	Over 5,000	155	14.2%
Religion influences family decisions	Yes	177	16.2%
	No	786	72.0%
	Unsure	129	11.8%
Receipt of 500+ child benefit	Yes	419	38.4%
	No	673	61.6%

Percentages may not sum to 100% due to rounding.

Most respondents reported a “good” (46.8%) or “average” (34.6%) subjective financial situation; 14.4% reported “very good” and 4.2% reported “rather bad” or “bad.” Household income per person was reported as 2,001–5,000 PLN/month by 48.4% of respondents, 1,001–2,000 PLN by 27.0%, more than 5,000 PLN by 14.2%, and up to 1,000 PLN by 10.3%. Religion was reported to influence family decisions by 16.2% of respondents, while 72.0% reported no such influence and 11.8% were unsure. Receipt of the 500+ child benefit was reported by 38.4% of respondents.

### 3.2 Reproductive intentions

**Figure 1:**
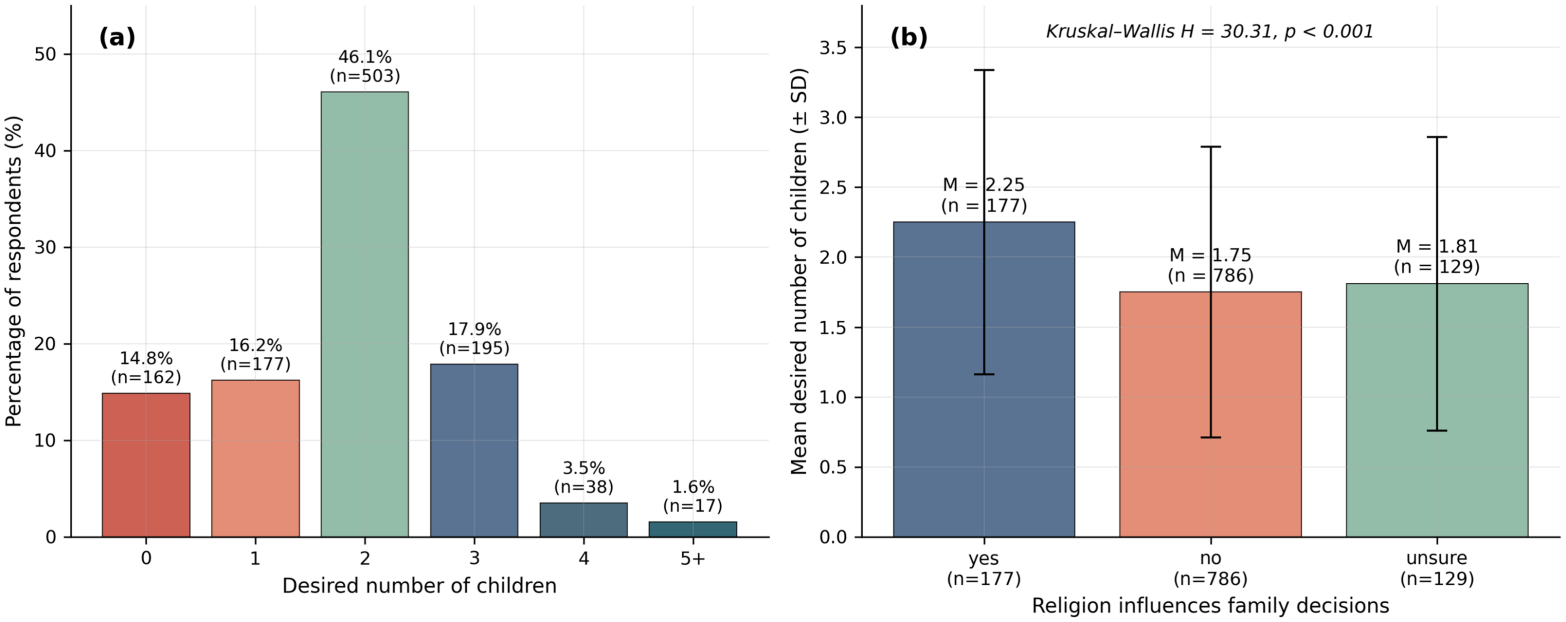
Distribution of the desired number of children in the sample (*N* = 1,092). Panel **(a)** overall distribution; Panel **(b)** mean desired number of children stratified by whether religion influences family decisions (Kruskal–Wallis H = 30.31, *p* < 0.001).

The mean desired number of children was 1.84 (SD 1.09), with a median of 2 (Table 2; Figure 1a). The modal value was 2 (46.1% of respondents), followed by 3 (17.9%), 1 (16.2%), 0 (“none”) (14.8%), 4 (3.5%) and 5 or more (1.6%). Just under one in seven respondents therefore expressed a desire for no children at all, while one in five wished for three or more — a polarised distribution that we return to in the discussion. The preferred family form was marriage for 79.9% of respondents, an informal partnership for 11.6, and 8.5% reported that they did not plan to start a family at all. Current contraceptive use was reported by 52.2% of respondents.

**Table 2:** Reproductive intentions, family form preferences and contextual factors (*N* = 1,092).

Variable	Category	*n*	%
Desired number of children	None	162	14.8%
	1	177	16.2%
	2	503	46.1%
	3	195	17.9%
	4	38	3.5%
	5 or more	17	1.6%
Mean (SD); median	1.84 (1.09); 2	—	—
Preferred family form	Marriage	872	79.9%
	Informal partnership	127	11.6%
	No plans to start a family	93	8.5%
Current contraceptive use	Yes	570	52.2%
	No	522	47.8%
Ukraine war influences fertility decisions	Yes	353	32.3%
	No	686	62.8%
	Missing	53	4.9%

When asked whether the war in Ukraine could influence their decisions about having children, 62.8% answered “no” and 32.3% answered “yes” (with 4.9% missing). Stratification by age (Table 3) showed a clear gradient: 28.7% of respondents aged 18–20 reported a perceived influence of the war, rising progressively to 39.1% among those over 40 (*χ*^2^ = 11.34; df = 4; *p* = 0.023). The pattern by place of residence was less monotonic but still significant (*χ*^2^ = 10.99; df = 4; *p* = 0.027), with the highest proportion reporting a perceived influence in cities of 50,001–150,000 inhabitants (42.0%) and the lowest in cities over 150,000 (26.7%).

**Table 3:** Perceived influence of the war in Ukraine on reproductive decisions, stratified by age.

Age group	No (n)	Yes (n)	Missing (n)	Total (n)	% Yes
18–20	253	103	3	359	28.7%
21–25	137	56	17	210	26.7%
26–30	74	44	8	126	34.9%
31–40	112	73	15	200	36.5%
Over 40	110	77	10	197	39.1%
Total	686	353	53	1,092	32.3%

Pearson *χ*^2^ = 11.34, df = 4, *p* = 0.023 (comparison of Yes vs other responses across age groups). Percentages computed as Yes divided by the total number of respondents in each age group, including those with missing data on this item.

### 3.3 Hierarchy of perceived barriers

**Figure 2:**
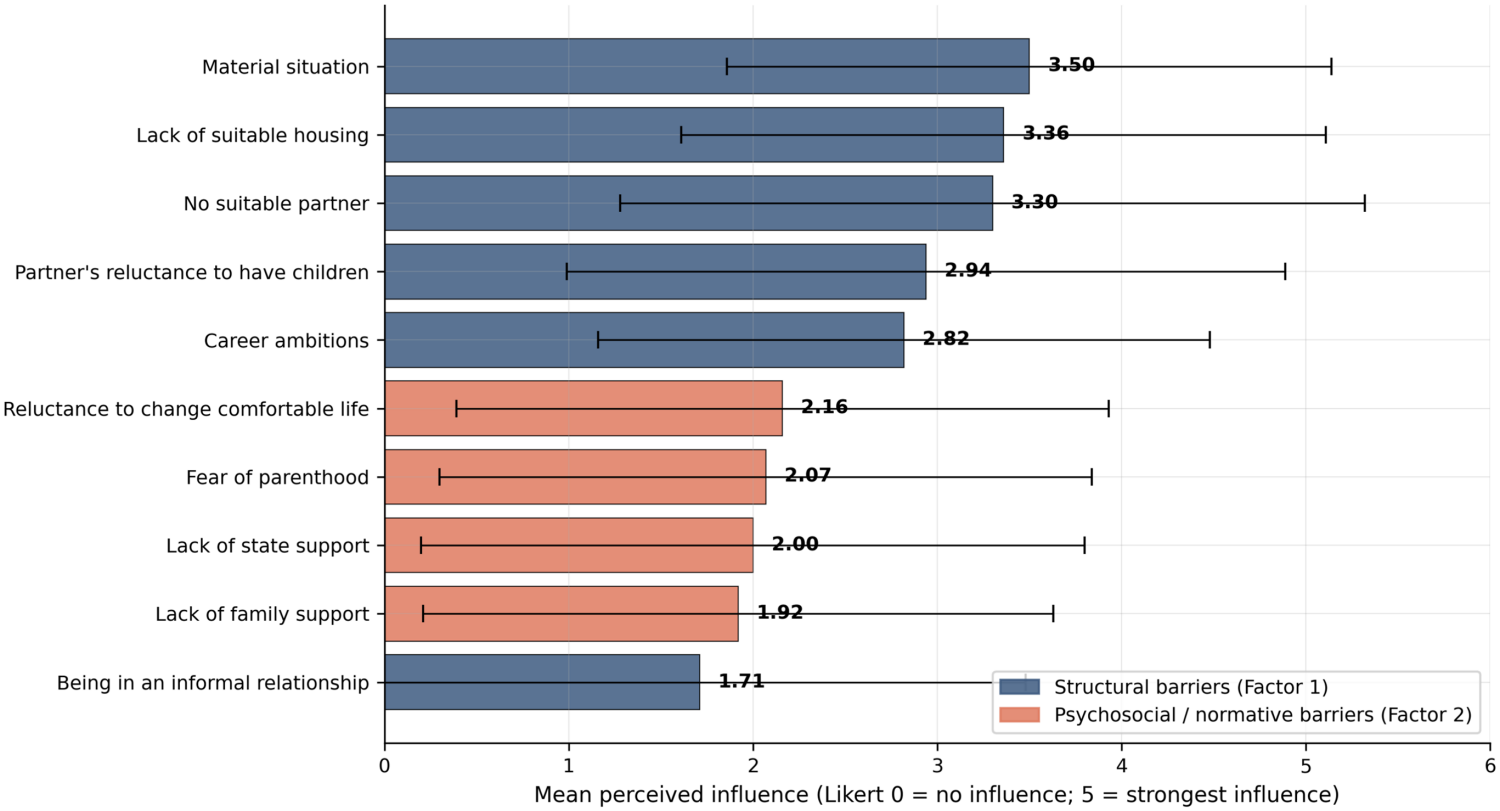
Hierarchy of perceived barriers to childbearing (Likert scale 0 = no influence to 5 = strongest influence; *N* = 1,092). Bars are colour-coded according to the two-factor solution: structural barriers (Factor 1) in blue and psychosocial-normative barriers (Factor 2) in orange. Means with standard deviations.

The 10 barrier items were rated by all respondents on the 0–5 Likert scale. The hierarchy of perceived influence is shown in Figure 2 and Table 4. The three highest-rated barriers were material situation (*M* = 3.50; SD 1.64), lack of suitable housing (*M* = 3.36; SD 1.75) and lack of a suitable partner (*M* = 3.30; SD 2.02). The next tier comprised partner’s reluctance to have children (*M* = 2.94; SD 1.95) and career ambitions (*M* = 2.82; SD 1.66). The lowest-rated barriers were lack of family support (*M* = 1.92; SD 1.71) and being in an informal relationship (*M* = 1.71; SD 1.77). Overall, material and residential conditions therefore emerged as the most salient perceived barriers, ahead of psychosocial and normative concerns.

**Table 4:** Hierarchy of perceived barriers to childbearing (Likert 0–5; *N* = 1,092).

Rank	Barrier	Mean	SD	Median
1	Material situation	3.50	1.64	4
2	Lack of suitable housing	3.36	1.75	4
3	Lack of a suitable partner	3.30	2.02	5
4	Partner’s reluctance to have children	2.94	1.95	3
5	Career ambitions	2.82	1.66	3
6	Reluctance to change one’s current life	2.16	1.77	2
7	Fear of parenthood	2.07	1.77	2
8	Lack of state support	2.00	1.80	2
9	Lack of family support	1.92	1.71	2
10	Being in an informal relationship	1.71	1.77	1

Likert scale: 0 = no influence; 5 = strongest influence.

### 3.4 Bivariate associations with desired number of children

Bivariate associations are summarised in Table 5. Older respondents reported a higher desired number of children (Spearman *ρ* = 0.18; *p* < 0.001). Educational attainment was significantly associated with desired number of children in the Kruskal–Wallis test (H = 25.90; *p* < 0.001), with respondents holding tertiary education reporting the highest mean (1.98) and respondents with only primary education the lowest (1.43). Urbanisation was negatively associated with desired number of children (*ρ* = −0.08; *p* = 0.012). Religion influencing family decisions was strongly associated with the outcome (Kruskal–Wallis H = 30.31; *p* < 0.001), with respondents reporting religious influence wanting on average 2.25 children compared with 1.75 among those reporting no such influence and 1.81 among those who were unsure (Figure 1b). Family of origin size was positively associated with desired number of children (ρ = 0.18; *p* < 0.001).

**Table 5:** Bivariate associations between selected predictors and the desired number of children.

Predictor	Test	Statistic	*p*-value
Age (categories 18–20 to >40, midpoints used)	Spearman	ρ = 0.18	< 0.001***
Educational attainment	Kruskal–Wallis	H = 25.90	< 0.001***
Place of residence (rural to city >150,000)	Spearman ordinal	ρ = −0.08	0.012*
Religion influences family decisions	Kruskal–Wallis	H = 30.31	< 0.001***
Subjective financial situation (1–5)	Spearman	ρ = 0.02	0.54
Receipt of 500+ child benefit	Mann–Whitney U	U = 124,202	< 0.001***
Family of origin size	Spearman	ρ = 0.18	< 0.001***
Ukraine war influences decisions	Mann–Whitney U	U = 132,542	0.65

Outcome: desired number of children (ordinal 0–5). ***p < 0.001; **p < 0.01; *p < 0.05.

Subjective financial situation showed no statistically significant association with desired number of children (ρ = 0.02; *p* = 0.54). Receipt of the 500+ child benefit, in contrast, was positively associated with the outcome in the bivariate Mann–Whitney U test (U = 124,202; *p* < 0.001), with respondents receiving the benefit reporting a higher mean desired number of children (1.99) than those not receiving it (1.74). Perceived influence of the Ukraine war showed no significant bivariate association with the outcome (U = 132,542; *p* = 0.65).

### 3.5 Adjusted multivariable analysis

**Figure 3:**
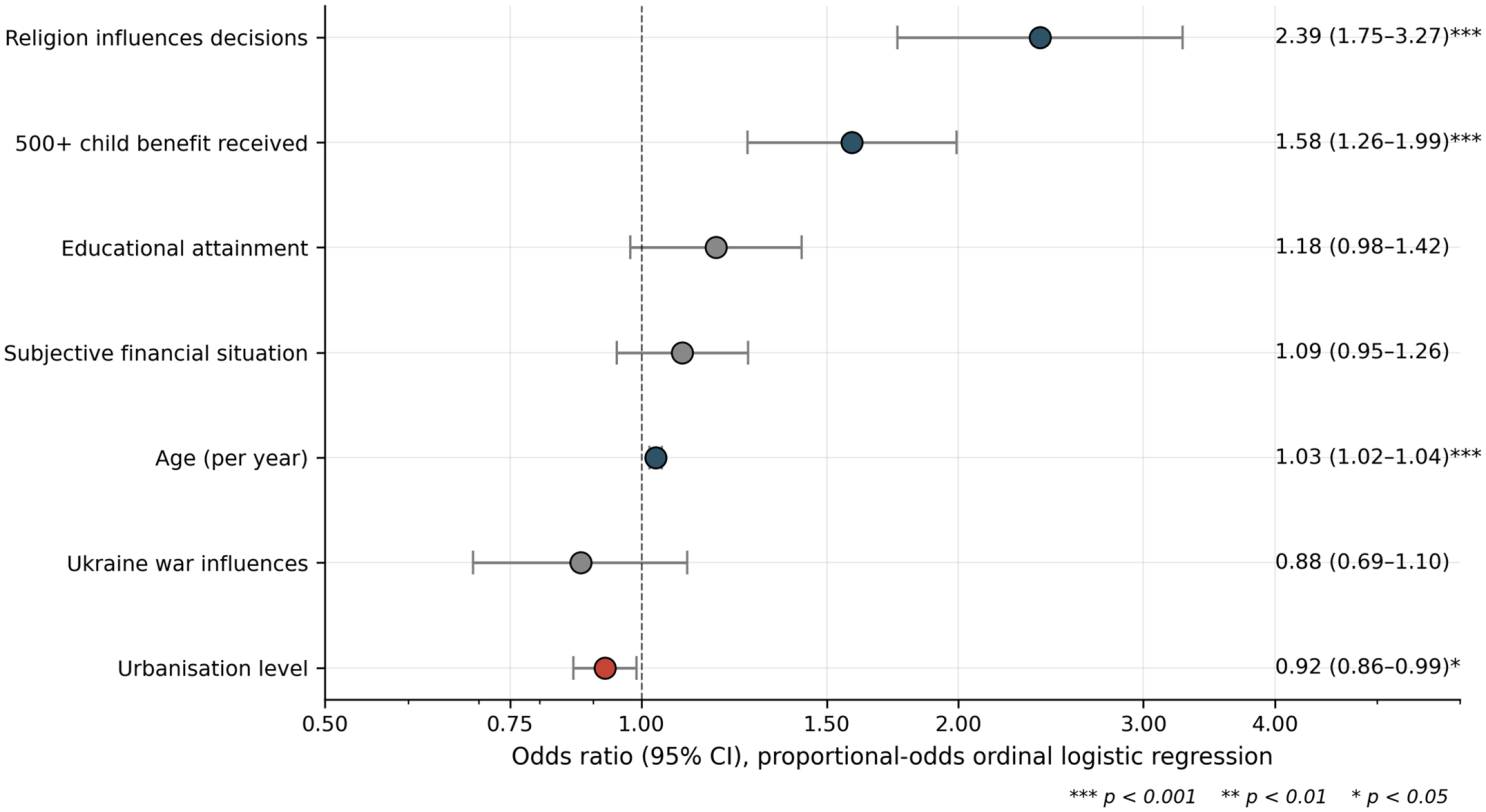
Adjusted odds ratios with 95% confidence intervals from the proportional-odds ordinal logistic regression (*n* = 1,092). Outcome: desired number of children. Predictors entered simultaneously. The dashed line marks an odds ratio of 1.00. ***p < 0.001; **p < 0.01; *p < 0.05.

In the proportional-odds ordinal logistic regression (*n* = 1,092 complete cases), four predictors remained statistically significant after adjustment for the others (Table 6; Figure 3). Religion influencing family decisions was the strongest adjusted predictor (OR = 2.39; 95% CI 1.75–3.27; *p* < 0.001), indicating that respondents reporting religious influence had more than twice the odds of being in a higher category of desired number of children. Receipt of the 500+ child benefit was a substantial and statistically significant adjusted predictor (OR = 1.58; 95% CI 1.26–1.99; *p* < 0.001). Age contributed a small but consistent adjusted effect (OR = 1.03 per year; 95% CI 1.02–1.04; *p* < 0.001), reflecting a roughly 30% increase in the odds of being in a higher outcome category for every decade of age. Urbanisation level was inversely associated with desired number of children after adjustment (OR = 0.92 per category; 95% CI 0.86–0.99; *p* = 0.023).

**Table 6:** Adjusted proportional-odds ordinal logistic regression (*n* = 1,092).

Predictor	OR	95% CI	*p*-value
Religion influences family decisions (yes vs. no)	2.39	1.75–3.27	< 0.001***
Receipt of 500+ child benefit (yes vs. no)	1.58	1.26–1.99	< 0.001***
Educational attainment (per category, 1–4)	1.18	0.98–1.42	0.088
Subjective financial situation (per level, 1–5)	1.09	0.95–1.26	0.226
Age (per year)	1.03	1.02–1.04	< 0.001***
Ukraine war influences decisions (yes vs. no)	0.88	0.69–1.10	0.262
Urbanisation level (per category, 1–5)	0.92	0.86–0.99	0.023*

Outcome: desired number of children, treated as ordinal (0, 1, 2, 3, 4, 5+). All predictors entered simultaneously. ***p < 0.001; **p < 0.01; *p < 0.05. OR > 1 indicates higher odds of being in a higher outcome category for a one-unit increase in the predictor.

Three predictors that had shown bivariate associations did not retain statistical significance after adjustment. Educational attainment was attenuated to a non-significant point estimate (OR = 1.18; 95% CI 0.98–1.42; *p* = 0.088). Subjective financial situation was non-significant in both bivariate and adjusted analyses (adjusted OR = 1.09; 95% CI 0.95–1.26; *p* = 0.23). Perceived influence of the Ukraine war was non-significant after adjustment (OR = 0.88; 95% CI 0.69–1.10; *p* = 0.26), indicating that the bivariate gradient by age category did not translate into an independent effect on the outcome.

### 3.6 Latent structure of perceived barriers

**Figure 4:**
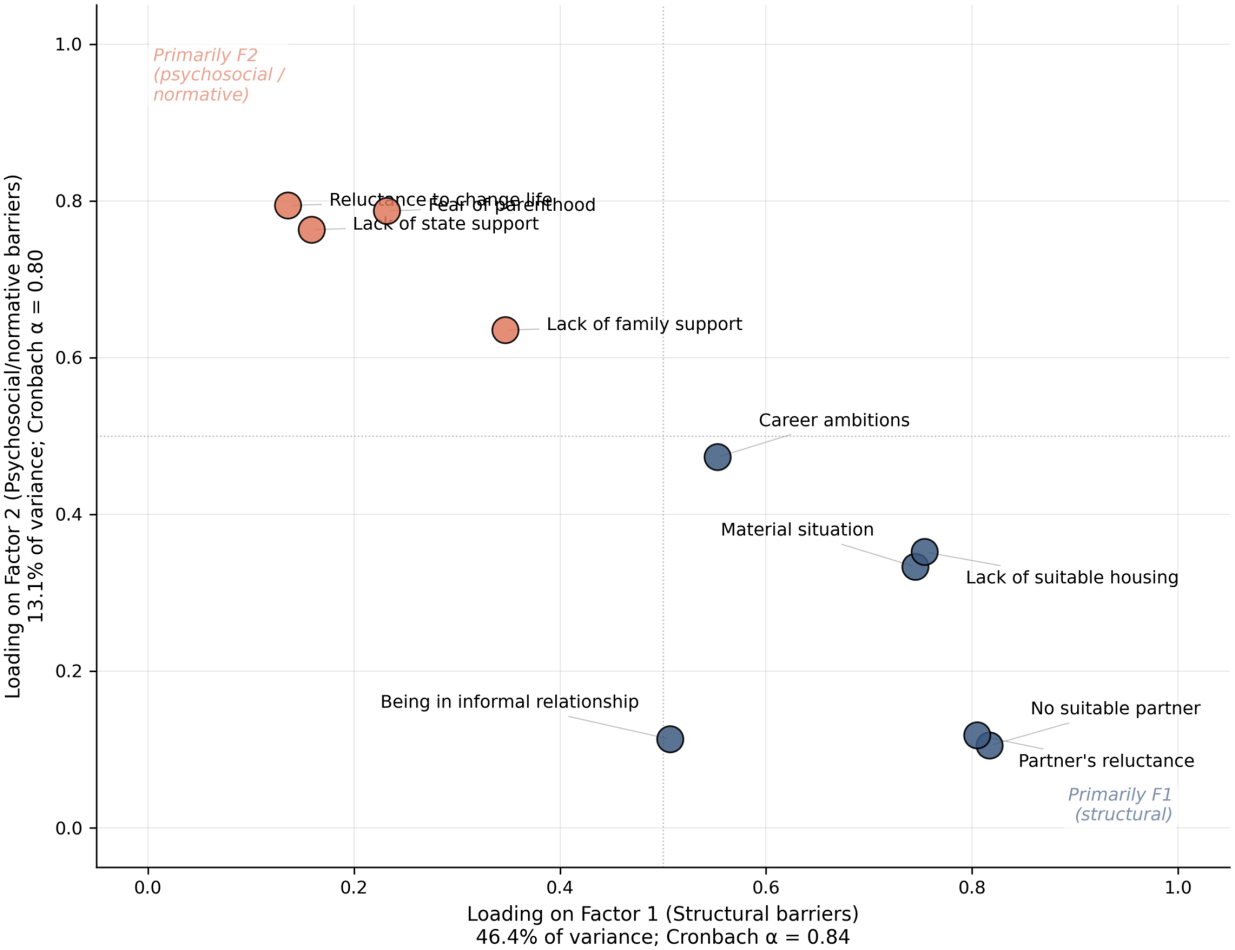
Two-factor loading map for the 10-item barrier instrument, after varimax rotation (*N* = 1,092; KMO = 0.86; Bartlett *p* < 0.001). Factor 1 (Structural barriers): 46.4% of variance, Cronbach *α* = 0.84. Factor 2 (Psychosocial-normative barriers): 13.1% of variance, Cronbach *α* = 0.80.

The 10-item barrier instrument was suitable for factor analysis (Bartlett’s test *χ*^2^ = 4,766.2; df = 45; *p* < 0.001; KMO = 0.86). Principal-component analysis with subsequent varimax rotation identified two interpretable factors with eigenvalues greater than 1, jointly explaining 59.5% of the variance (Table 7; Figure 4). The first factor (eigenvalue 4.64; 46.4% of variance; Cronbach *α* = 0.84) had its highest loadings on the items “lack of a suitable partner” (loading 0.82), “partner’s reluctance to have children” (0.81), “lack of suitable housing” (0.75), “material situation” (0.75), “career ambitions” (0.55) and “being in an informal relationship” (0.51). We labelled this factor structural barriers, reflecting the fact that all six items capture potentially modifiable conditions of life that fall within the domain of public policy or material reality.

**Table 7:** Varimax-rotated factor loadings for the 10-item barrier instrument (*N* = 1,092).

Item	F1 (structural)	F2 (psychosocial-normative)	Primary loading
Lack of a suitable partner	0.82	0.10	F1
Partner’s reluctance to have children	0.81	0.12	F1
Lack of suitable housing	0.75	0.35	F1
Material situation	0.75	0.33	F1
Career ambitions	0.55	0.47	F1 (cross)
Being in an informal relationship	0.51	0.11	F1
Reluctance to change one’s current life	0.14	0.79	F2
Fear of parenthood	0.23	0.79	F2
Lack of state support	0.16	0.76	F2
Lack of family support	0.35	0.64	F2

KMO = 0.86; Bartlett’s test *χ*^2^ = 4,766.2, df = 45, *p* < 0.001. Two factors retained (eigenvalues > 1) accounting jointly for 59.5% of the variance (F1: 46.4%; F2: 13.1%). Cronbach *α*: F1 = 0.84; F2 = 0.80. Loadings ≥ 0.50 considered salient.

The second factor (eigenvalue 1.31; 13.1% of variance; Cronbach α = 0.80) had its highest loadings on “reluctance to change one’s current comfortable life” (0.79), “fear of parenthood” (0.79), “lack of state support” (0.76) and “lack of family support” (0.64). We labelled this factor psychosocial-normative barriers, reflecting the fact that these items capture psychological orientations and perceived social-support deficits that operate in the cultural and normative domain rather than through directly modifiable material conditions. The two-factor solution showed a clean separation in the loading map (Figure 4): six items loaded primarily on Factor 1 with negligible secondary loadings, four items loaded primarily on Factor 2, and only “career ambitions” showed a substantial cross-loading consistent with its dual character as both a material-structural and a personal-life-course concern.

## 4 Discussion

### 4.1 Principal findings

In our cross-sectional sample of 1,092 respondents in the Greater Poland Voivodeship, the desired number of children is low and unequally distributed: nearly half of respondents wish for two children, 14.8% want none, and only 22.9% want three or more. The mean of 1.84 sits above the regional period total fertility rate of 1.17 in 2024 (6)—a gap that itself merits comment, since it reflects the established intention–behaviour discrepancy in low-fertility settings. In the adjusted multivariable model, religion, receipt of the 500+ child benefit, age and the rural–urban gradient remained independent predictors of reproductive intentions; subjective financial situation, educational attainment and the perceived influence of the war in Ukraine did not. Exploratory factor analysis of the 10 barrier items revealed a stable two-factor structure that separates structural barriers—partner availability, material situation, housing, career, informal relationship—from psychosocial-normative barriers—fear of parenthood, lack of family and state support, reluctance to change one’s life. The two-factor solution accounted for nearly 60% of the variance and showed acceptable internal consistency, supporting an interpretation in which different families of barriers operate through distinct mechanisms.

These results should be read alongside the companion regional analysis of administrative birth records (6), which documented a 31.9% decline in live births in Greater Poland between the 2017 peak and 2023. The convergence of evidence at two complementary levels of analysis—population-level trends and individual-level intentions—is the central methodological contribution of this paper: an individual-level account that mirrors and partially explains the aggregate trajectory.

### 4.2 Interpretation in the European context

The polarised distribution of desired number of children, with substantial proportions wishing for either zero or three-plus, fits a wider European pattern. Beaujouan (12) traced the resurgence of late parenthood across low-fertility countries and showed that the proportion of women with no completed births has risen substantially in cohorts entering reproductive ages from the 1970s onwards. Lazzari et al. (2) demonstrated that perceptions of the optimal reproductive age window have shifted towards later parenthood across 21 European countries, with consequences for period fertility indicators. The 14.8% of our respondents wishing for no children fits the postponement-childlessness pattern documented in those studies. The 22.9% wishing for three or more children—concentrated, as our adjusted model shows, among older, more religious and more rural respondents—points to a parallel persistence of larger family ideals in particular subgroups.

Religion was the strongest adjusted predictor of higher reproductive intentions in our analysis. The finding aligns with several streams of European evidence on the persistence of normative influences on family-formation decisions. Esping-Andersen and Billari (11) argued that fertility outcomes in low-fertility settings reflect the diffusion of gender-egalitarian norms, with religion functioning as one of the cultural anchors that sustain larger family ideals in some subpopulations. The size of the adjusted effect we observed—more than a doubling of the odds of being in a higher outcome category—is striking, and merits replication in other Polish regions.

### 4.3 Subjective financial situation and the 500+ benefit

Two findings concerning economic factors deserve careful interpretation. The first is the absence of any significant association between subjective financial situation and desired number of children, in either the bivariate or the adjusted analysis. This pattern is consistent with European evidence from persistent low-fertility settings: Matysiak et al. (15) showed that the relationship between the business cycle and fertility is heterogeneous and frequently non-significant in such settings. The present finding extends that pattern to subjective economic perceptions at the individual level. It also fits the absence of a positive ecological association between rising regional per-capita income and births in Greater Poland (6).

The second is the substantial adjusted association between receipt of the 500+ child benefit and higher desired number of children (OR = 1.58; 95% CI 1.26–1.99; *p* < 0.001). This finding requires careful interpretation, because the cross-sectional design and the nature of the outcome—desired rather than achieved fertility—preclude a causal claim. The most plausible interpretation is reverse causation or selection: respondents who already have children are eligible for the benefit, and they are more likely to want additional children than those who have none. The result is therefore best read as a correlate of family situation, not as evidence of a behavioural effect of the transfer programme. Read in this light, the finding does not contradict the conclusions of Bergsvik et al. (13), who in a systematic review of (quasi-)experimental literature concluded that increased cash transfers tend to have only temporary effects on fertility, while childcare expansions are associated with more sustained increases. If anything, it underlines that desired-fertility responses to transfer receipt should not be over-interpreted in cross-sectional designs.

### 4.4 The structural/psychosocial-normative dichotomy

The factor analysis identified two latent dimensions that correspond to qualitatively different families of barriers and have direct implications for policy interpretation. The first factor, structural barriers, encompasses material situation, housing, partner availability, partner’s preferences and career ambitions. These are barriers that, at least in principle, are amenable to intervention through housing policy, family-friendly labour-market arrangements, and public support for partner-formation conditions (such as affordable rental housing or childcare expansion). The second factor, psychosocial-normative barriers, encompasses fear of parenthood, perceived lack of family and state support, and reluctance to change one’s current comfortable life. These barriers operate in the cultural and normative domain. They are less amenable to direct policy intervention; they may respond more slowly, and through different channels — changing intergenerational relations, the perceived legitimacy of family-related institutions, broader cultural framings of parenthood (9).

The implication is that no single policy lever can address the full spectrum of perceived barriers. Material and housing supports may directly affect Factor 1. The more diffuse Factor 2 calls for sustained engagement with cultural narratives, perceived security and social-support infrastructures.

### 4.5 The Ukraine war, age and uncertainty

A clear bivariate gradient emerged by age in the perceived influence of the war in Ukraine on reproductive decisions—from 28.7% among 18–20-year-olds to 39.1% among those over 40. The gradient is worth noting on its own, but it does not translate into an independent effect on desired number of children once other variables are controlled. One reading is that older respondents are more likely to interpret reproductive decisions through a geopolitical risk lens. The larger effects of age and family situation on desired-fertility outcomes simply dominate this perception in the multivariable model. Vignoli et al. (9) showed that perceived uncertainty is a meaningful determinant of fertility postponement, and our finding is consistent with that view at the level of perception, while showing that perception alone does not displace the structural predictors of intentions.

### 4.6 Implications for public health policy

Several implications for public health and family policy in Central Europe follow from this work. Regional health-needs assessment should incorporate individual-level evidence on reproductive intentions alongside aggregate vital-statistics data, since the two levels of analysis do not always agree on policy implications. The structural/psychosocial-normative dichotomy uncovered by the factor analysis indicates that policy mixes targeting only one dimension are unlikely to be sufficient: housing and labour-market interventions address structural barriers, while sustained engagement with family-related norms and support infrastructures is needed for the psychosocial-normative dimension. The absence of an adjusted association between subjective financial situation and reproductive intentions, combined with the cross-sectional positive association between 500+ receipt and intentions, points to a further conclusion—that family policy framed purely in terms of cash transfers is incomplete. Our findings therefore lend individual-level support to the public health planning conclusion drawn from the regional birth-trend analysis (6): demographic monitoring should be treated as an integral component of regional public health policy, supporting evidence-based responses to the longer-term restructuring of population needs.

## 5 Strengths and limitations

The study has several methodological strengths. The sample of 1,092 respondents is substantial for a regional survey of fertility intentions and provides reasonable power to detect moderate effects in the adjusted ordinal model. The questionnaire was based on an instrument piloted earlier by the same research group (*n* = 419), supporting its content validity. The barrier instrument showed strong sampling adequacy (KMO = 0.86) and identified two interpretable factors with acceptable internal consistency (Cronbach *α* = 0.84 and 0.80). The combination of bivariate and multivariable adjusted analyses, together with exploratory factor analysis, provides convergent evidence for the principal findings.

Several limitations should also be acknowledged. The cross-sectional design precludes causal inference. In particular, the association between 500+ receipt and desired number of children should be read as a correlate of family situation, not as evidence of a behavioural effect of the transfer programme. Recruitment through mixed online channels produced a convenience sample that cannot be assumed to be representative of the Greater Poland population; the over-representation of younger respondents and respondents currently in education is consistent with this concern. The desired number of children is a stated intention rather than completed fertility, and the gap between intentions and outcomes is well documented in the European literature (12). Self-report bias may have affected sensitive items such as religion, contraception and alcohol use, and social-desirability bias cannot be excluded for normatively charged items such as the influence of the war in Ukraine. The barrier instrument used a 0–5 scale; while the high KMO and the clean two-factor structure support its measurement properties in this sample, replication in other Polish and Central European regions would strengthen external validity. The analysis treated educational attainment and place of residence as ordinal—a simplification of the underlying categorical structure adopted to preserve the interpretability of the proportional-odds model.

## 6 Future research

Several directions for future work follow from this analysis. Longitudinal designs that link stated intentions to subsequent reproductive outcomes would help quantify the intention–behaviour gap in Central European settings. Comparative regional analyses across Polish voivodeships and other Central and Eastern European regions would test the external validity of the structural / psychosocial-normative dichotomy and identify the contextual factors that mediate it. Mixed-methods designs combining survey data with qualitative work could illuminate the cultural and normative pathways through which Factor 2 operates, and inform the design of public health interventions aimed at supporting reproductive choices.

## 7 Conclusion

Fertility intentions in this Central European regional sample are shaped by a heterogeneous mix of structural, normative and life-course factors that cannot be reduced to material conditions alone. Religion, age, urbanisation level and family situation (proxied by 500+ benefit receipt) emerged as independent adjusted predictors of higher desired number of children. Subjective financial situation, educational attainment and the perceived influence of the Ukraine war did not. Perceived barriers to childbearing were reducible to two interpretable dimensions—structural and psychosocial-normative—with direct implications for the design of public health and family policy. Together with the companion analysis of regional birth trends (6), these findings support a multi-dimensional approach to demographic monitoring and to the integration of individual-level evidence into regional health and social services planning.

## Data Availability

The original contributions presented in the study are included in the article/Supplementary material, further inquiries can be directed to the corresponding author.
