# Supplementary material for: Determinants of fertility intentions in a low-fertility region of Central Europe: a cross-sectional survey of 1,092 respondents in the Greater Poland Voivodeship
Source: Front Public Health. 2026 Jul 7;14:1872501. doi: 10.3389/fpubh.2026.1872501 (PMC13384937; doi:10.3389/fpubh.2026.1872501)
Supplement: Supplementary file 1 [file Data_Sheet_1.docx]

**Supplementary Materials**

*Determinants of childbearing intentions in a low-fertility region of Central Europe: a cross-sectional survey of 1,092 respondents in the Greater Poland Voivodeship*

Ciborek A., Chęcińska-Maciejewska Z., Nowak A., Krauss H.

**Supplementary Table S3. Comparison of the achieved sample with the demographic structure of the Greater Poland Voivodeship**

The table compares the achieved sample (n = 1,092; cross-sectional online survey, March 2023 – March 2024) with corresponding regional population reference values for the Greater Poland Voivodeship, drawn primarily from the National Population and Housing Census 2021 (Statistics Poland; Statistical Office in Poznań) and regional demographic situation reports for 2023. The comparison is presented for variables for which a direct census-level reference is available, namely age category, educational attainment and place of residence on the rural–urban gradient. Population reference values refer to the adult population (18+ years) of the Greater Poland Voivodeship.

**Panel A — Age category**

| Age category | n in sample | % in sample | % in regional adult population (18+) | Direction of bias |
| --- | --- | --- | --- | --- |
| 18–20 years | 359 | 32.9% | ~4% | Strong over-representation |
| 21–25 years | 210 | 19.2% | ~7% | Over-representation |
| 26–30 years | 126 | 11.5% | ~8% | Close to reference |
| 31–40 years | 200 | 18.3% | ~16% | Close to reference |
| Over 40 years | 197 | 18.0% | ~65% | Strong under-representation |

*Sources: sample (this study); regional reference — Statistics Poland, age structure of the population of the Greater Poland Voivodeship, 2023. Reference percentages computed for the adult population (18+ years) and rounded.*

**Panel B — Educational attainment**

| Educational attainment | n in sample | % in sample | % in regional adult population (18+) | Direction of bias |
| --- | --- | --- | --- | --- |
| Tertiary (higher) | 375 | 34.3% | 23.5% | Over-representation |
| Secondary (general + vocational) | 351 | 32.1% | 30.8% | Slight over-representation |
| Currently studying | 284 | 26.0% | n.a. (separate category) | Specific to study sample |
| Vocational (basic) | 42 | 3.8% | 25.0% | Strong under-representation |
| Primary or below | 40 | 3.7% | 14.7% | Strong under-representation |

*Sources: sample (this study); regional reference — National Population and Housing Census 2021, Statistics Poland, educational attainment of the population of the Greater Poland Voivodeship aged 13+ years. Note: the National Census does not contain a separate category for respondents who declare ongoing education without yet holding a tertiary degree; in the present sample, respondents reporting 'currently studying' were predominantly young adults enrolled in higher education and have been treated alongside 'secondary' in the proportional-odds analysis (see manuscript Section 2.3).*

**Panel C — Place of residence (rural–urban gradient)**

| Place of residence | n in sample | % in sample | % in regional population | Direction of bias |
| --- | --- | --- | --- | --- |
| Rural area | 358 | 32.8% | ~47% | Under-representation |
| Town ≤ 20,000 | 159 | 14.6% | ~10% | Slight over-representation |
| Town 20,001–50,000 | 147 | 13.5% | ~13% | Close to reference |
| City 50,001–150,000 | 143 | 13.1% | ~9% | Slight over-representation |
| City > 150,000 | 285 | 26.1% | ~21% | Slight over-representation |

*Sources: sample (this study); regional reference — Statistics Poland, distribution of the population of the Greater Poland Voivodeship by place of residence (urban/rural). Reference percentages for sub-categories of urban settlements are approximations based on regional settlement structure data and may vary slightly across reporting years.*

**Note on sex**

The survey instrument did not include a structured item recording the respondent's biological sex; all questions referred to potential parenthood in mixed language form (Pan/Pani — 'you'). Consequently, the achieved sample could not be cross-tabulated against the regional population by sex. Future replications of the survey are encouraged to add a structured sex item to enable formal weighting and representativeness analysis.

**Overall interpretive note**

The comparison documents a clear over-representation of young adults (18–25 years), respondents in tertiary education or currently enrolled in tertiary education, and respondents from urban areas; a strong under-representation of adults aged over 40 years, respondents with vocational and primary education, and respondents from rural areas. This pattern is consistent with the known demographic profile of online survey participants, particularly for instruments disseminated through social media and university channels. The selection bias should be borne in mind when interpreting all bivariate and adjusted associations reported in the main manuscript (Sections 3 and 4), and when generalising the findings to the regional population as a whole.

**Supplementary Table S4. Diagnostic tests of the proportional-odds assumption and partial-proportional-odds sensitivity analysis**

The proportional-odds (PO) assumption in the main ordinal logistic regression model (manuscript Section 2.4, Table 5) was assessed by the Brant test (Brant, 1990), which compares the regression coefficients estimated across all binary thresholds of the ordinal outcome (here, J−1 = 5 thresholds for the desired number of children scored 0 to 5). Significant departures from proportionality indicate that the effect of a given predictor differs across response thresholds — for example, that a predictor may have a stronger effect on the transition from 'no children' to 'at least one child' than on transitions to higher parities. Where the assumption was violated, threshold-specific effects were estimated by means of a partial-proportional-odds (generalised ordered logit) sensitivity analysis, in which separate odds ratios are reported per threshold.

**Panel A — Per-predictor and global Brant test of the proportional-odds assumption (n = 1,039, complete cases on all predictors including the Ukraine-war item; see note below)**

| Predictor | Brant χ² | df | p-value | Interpretation |
| --- | --- | --- | --- | --- |
| Age (per year, continuous) | 3.57 | 4 | 0.4679 | n.s. |
| Educational attainment (ordinal) | 17.93 | 4 | 0.0013 | ** |
| Urbanisation level (ordinal) | 10.09 | 4 | 0.0389 | * |
| Religion influencing family decisions (binary) | 9.71 | 4 | 0.0455 | * |
| Subjective financial situation (ordinal) | 1.26 | 4 | 0.8683 | n.s. |
| Perceived influence of Ukraine war (binary) | 3.37 | 4 | 0.4973 | n.s. |
| Receipt of 500+ child benefit (binary) | 3.41 | 4 | 0.4920 | n.s. |
| Global (sum across predictors) | 49.34 | 28 | 0.0076 | ** |

*Brant test of the proportional-odds assumption. The test was implemented on the model used in Table 5 of the main manuscript. Note on sample sizes: the Brant test (Panel A) was performed on the complete-case sample (n = 1,039) restricted to respondents who answered the Ukraine-war item, in order to avoid imputation in a diagnostic procedure; the model fit indices (Panel B) and the partial-proportional-odds analysis (Panel C) follow the convention adopted in the main manuscript and replace missing responses on this single item with the modal "no" value (n = 1,092 analysed). The two estimation strategies yield directionally identical conclusions. Significance markers: n.s. = not significant; * p < 0.05; ** p < 0.01; *** p < 0.001. Educational attainment (p = 0.001), urbanisation level (p = 0.039) and religion (p = 0.046) show statistically significant departures from the PO assumption, motivating the threshold-specific analysis presented in Panel C.*

**Panel B — Model fit and goodness-of-fit statistics**

| Statistic | Value | Note |
| --- | --- | --- |
| Number of observations (complete cases) | 1,092 |  |
| Number of predictors | 7 |  |
| Log-likelihood (full model) | -1509.67 |  |
| Log-likelihood (null model) | -1555.42 |  |
| Likelihood-ratio χ² (vs. null) | 91.51 | df = 7, p < 0.001 |
| McFadden pseudo-R² | 0.0294 | Indicates modest predictive contribution typical of survey-based fertility models |
| Akaike Information Criterion (AIC) | 3043.34 |  |
| AIC — sensitivity model with age as categorical dummies | 3041.89 | ΔAIC = -1.45 (lower = better) |
| McFadden pseudo-R² — age-dummies model | 0.0318 | Comparable fit; conclusions unchanged |

*Model fit and sensitivity analysis. The sensitivity model replaced the continuous midpoint coding of age (values 19, 23, 28, 35, 45) with four dummy variables for age categories (reference = 18–20 years). Slightly improved fit (lower AIC) with categorical age does not alter the direction or interpretation of any other predictor; the midpoint specification is retained in the primary model for parsimony and interpretability of the per-year odds ratio.*

**Panel C — Partial-proportional-odds sensitivity: per-threshold odds ratios for the three predictors flagged by the Brant test**

For educational attainment, urbanisation and religion — the three predictors for which the PO assumption was rejected — the table below reports odds ratios from separate binary logistic regressions predicting (desired number of children > j) for j = 0, 1, 2, 3. The threshold for j = 4 (i.e. y > 4) was not estimated because only 17 respondents in the sample reported a desired number of children of five or more, yielding insufficient cases on the positive side of the threshold for a stable estimate. All four binary models adjusted for the full set of covariates from the main model.

| Predictor / Threshold | OR | 95% CI | p-value |  |
| --- | --- | --- | --- | --- |
| Educational attainment |  |  |  |  |
| y > 0 (any children vs. none) | 1.630 | [1.261, 2.107] | < 0.001 | *** |
| y > 1 (≥ 2 vs. ≤ 1) | 1.270 | [1.026, 1.571] | 0.0283 | * |
| y > 2 (≥ 3 vs. ≤ 2) | 0.968 | [0.760, 1.234] | 0.7944 |  |
| y > 3 (≥ 4 vs. ≤ 3) | 0.686 | [0.455, 1.032] | 0.0707 |  |
| Urbanisation level |  |  |  |  |
| y > 0 (any children vs. none) | 0.988 | [0.888, 1.099] | 0.8246 |  |
| y > 1 (≥ 2 vs. ≤ 1) | 0.895 | [0.824, 0.972] | 0.0087 | ** |
| y > 2 (≥ 3 vs. ≤ 2) | 0.921 | [0.840, 1.011] | 0.0842 |  |
| y > 3 (≥ 4 vs. ≤ 3) | 1.007 | [0.847, 1.198] | 0.9352 |  |
| Religion influencing family decisions |  |  |  |  |
| y > 0 (any children vs. none) | 1.801 | [1.054, 3.080] | 0.0315 | * |
| y > 1 (≥ 2 vs. ≤ 1) | 1.823 | [1.228, 2.707] | 0.0029 | ** |
| y > 2 (≥ 3 vs. ≤ 2) | 2.877 | [2.022, 4.093] | < 0.001 | *** |
| y > 3 (≥ 4 vs. ≤ 3) | 3.844 | [2.133, 6.925] | < 0.001 | *** |

*Partial-proportional-odds (generalised ordered logit) sensitivity analysis. For predictors flagged by the Brant test, separate binary logistic regressions were fit for each threshold of the ordinal outcome. Results show that the effect of religion increases progressively across thresholds (OR rising from 1.80 at the lowest threshold to 3.84 at the highest), consistent with the interpretation that religion is more strongly associated with intentions for higher-order parities. The effect of educational attainment is concentrated at the lowest threshold (transition from no children to at least one), consistent with literature suggesting that educational attainment primarily shapes the decision to enter parenthood rather than completed family size. The urbanisation effect appears most consistent at the middle thresholds. These threshold-specific patterns extend, but do not contradict, the proportional-odds estimates reported in Table 5 of the main manuscript.*

**Supplementary Table S5. Full rotated component matrix from exploratory factor analysis (varimax rotation) of the 10-item barrier instrument**

The table below reports the complete rotated component matrix from the exploratory factor analysis described in Section 2.4 of the main manuscript. The factor extraction was performed by principal-component analysis (PCA) on the 10-item Likert barrier instrument (0–5 scale). The Kaiser criterion (eigenvalue greater than 1) and inspection of the scree plot (Supplementary Figure S1) supported retention of two factors, jointly explaining 59.5% of the variance. The two factors were subsequently rotated using varimax rotation to obtain an orthogonal solution with maximally interpretable loadings. Sampling adequacy (KMO = 0.865) and Bartlett's test of sphericity (χ² = 4,766.2; df = 45; p < 0.001) supported the suitability of the data for factor analysis.

| Barrier item | F1 — Structural | F2 — Psychosocial-normative | Primary loading |
| --- | --- | --- | --- |
| B1. Lack of a suitable partner | 0.818 | 0.105 | F1 (Structural) |
| B2. Being in an informal relationship | 0.508 | 0.113 | F1 (Structural) |
| B3. Partner reluctance to have children | 0.805 | 0.118 | F1 (Structural) |
| B4. Material situation | 0.745 | 0.333 | F1 (Structural) |
| B5. Career ambitions | 0.554 | 0.473 | F1 (Structural) \| cross-loading |
| B6. Lack of suitable housing | 0.755 | 0.352 | F1 (Structural) |
| B7. Fear of parenthood | 0.232 | 0.788 | F2 (Psychosocial-normative) |
| B8. Lack of family support | 0.347 | 0.635 | F2 (Psychosocial-normative) |
| B9. Lack of state support | 0.159 | 0.763 | F2 (Psychosocial-normative) |
| B10. Reluctance to change current comfortable life | 0.136 | 0.794 | F2 (Psychosocial-normative) |

*Rotated factor loadings (varimax rotation). Cronbach's α: Factor 1 = 0.837; Factor 2 = 0.796. Variance explained after rotation: Factor 1 = 32.24%; Factor 2 = 27.34%; jointly = 59.58%. Item B5 ('Career ambitions') shows a substantial cross-loading on both factors, consistent with its dual character as both a material-structural concern (career-related material constraints) and a personal-life-course preference (autonomy and self-realisation). All other items load primarily on a single factor with secondary loadings below the conventional 0.40 threshold.*

**Eigenvalues and percentage of variance explained — all 10 components**

| Component | Eigenvalue | % of variance | Cumulative % | Status |
| --- | --- | --- | --- | --- |
| PC1 | 4.644 | 46.40% | 46.40% | Retained |
| PC2 | 1.314 | 13.12% | 59.52% | Retained |
| PC3 | 0.918 | 9.17% | 68.69% |  |
| PC4 | 0.749 | 7.48% | 76.17% |  |
| PC5 | 0.615 | 6.15% | 82.32% |  |
| PC6 | 0.493 | 4.92% | 87.24% |  |
| PC7 | 0.372 | 3.71% | 90.96% |  |
| PC8 | 0.348 | 3.47% | 94.43% |  |
| PC9 | 0.312 | 3.12% | 97.55% |  |
| PC10 | 0.245 | 2.45% | 100.00% |  |

*Eigenvalues from the unrotated principal-component decomposition. Two components were retained on the basis of the Kaiser criterion (eigenvalue greater than 1) and inspection of the scree plot (Supplementary Figure S1).*

**Supplementary Figure S1. Scree plot for the principal-component analysis of the 10-item barrier instrument**

The scree plot shows the eigenvalues from the unrotated principal-component decomposition of the 10 barrier items. The first two components have eigenvalues clearly above the Kaiser threshold of 1 (4.64 and 1.31, respectively); the third component falls just below this threshold (0.92), and subsequent components show a smooth decay. The 'elbow' of the curve at the third component supports the retention of two factors in the final solution.


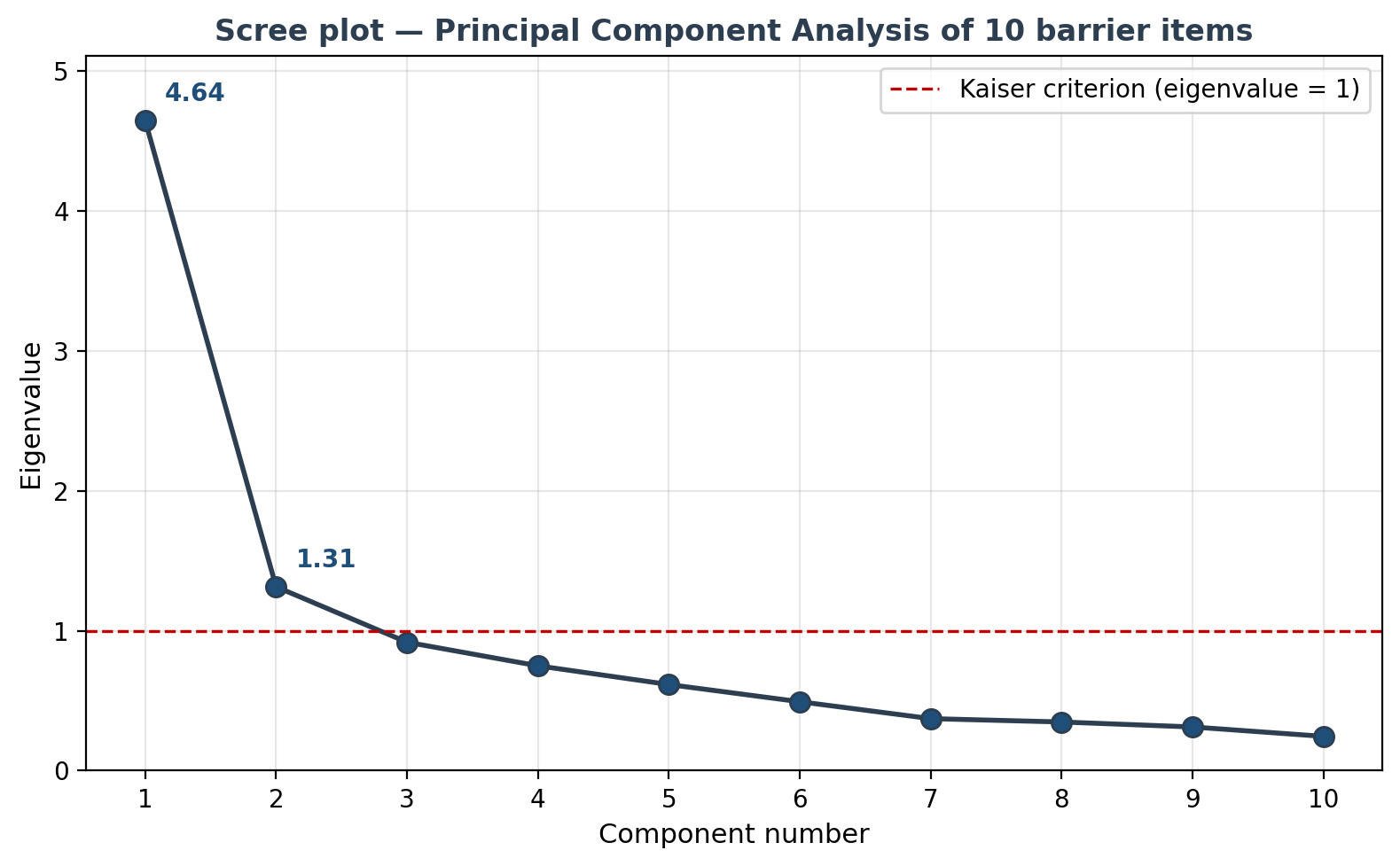


*Figure S1. Scree plot of eigenvalues from the principal-component analysis of the 10-item perceived-barrier instrument. The dashed red horizontal line marks the Kaiser criterion (eigenvalue = 1); components with eigenvalues above this line were retained.*

**Supplementary Figure S2. Rotated factor loadings — bar chart**

The bar chart summarises the rotated factor loadings of the 10 barrier items on the two retained factors. Items B1–B6 load primarily on Factor 1 (Structural barriers), while items B7–B10 load primarily on Factor 2 (Psychosocial-normative barriers). The two dotted red vertical lines mark the conventional ±0.40 threshold for interpretive significance of factor loadings.


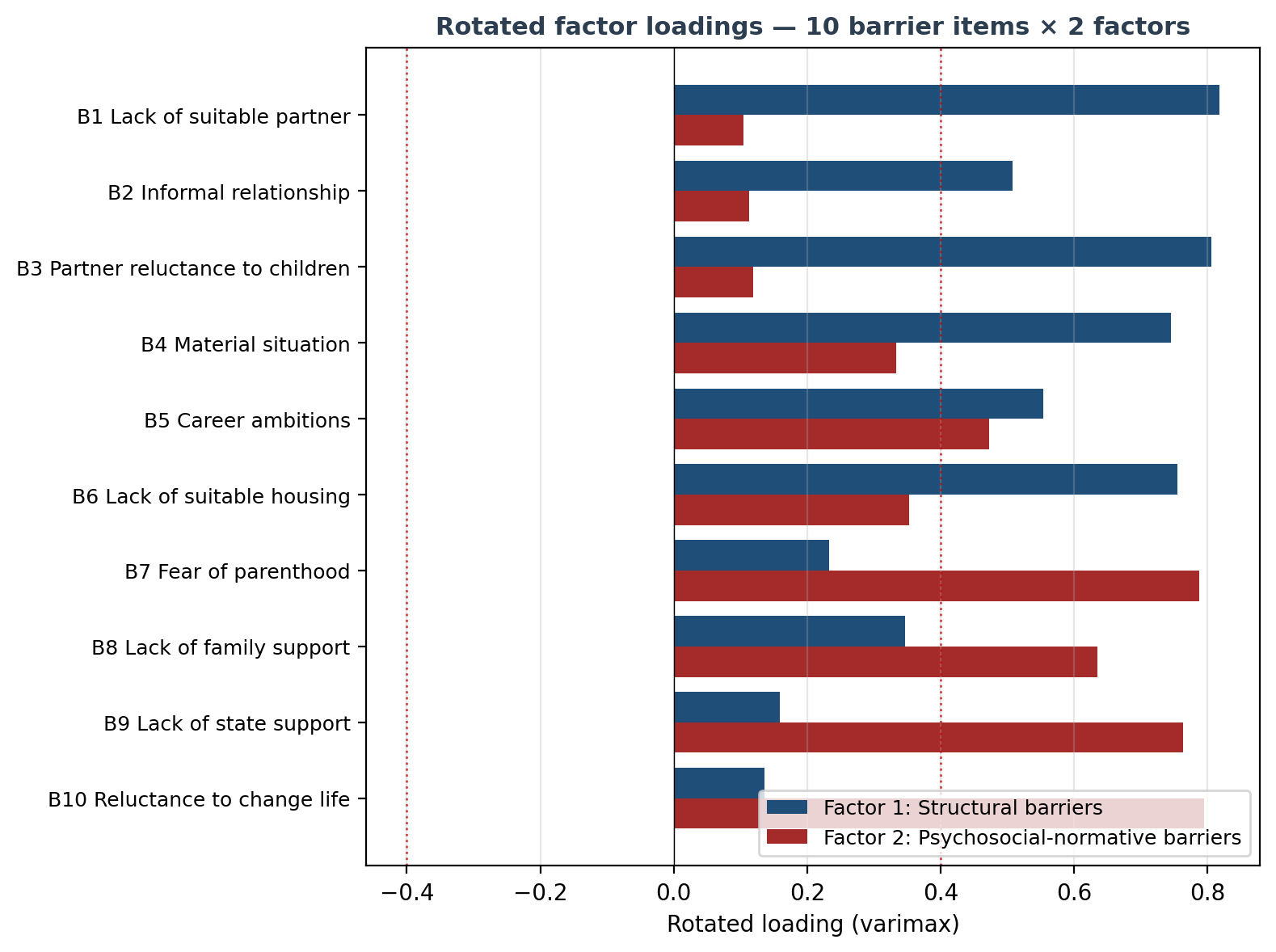


*Figure S2. Rotated factor loadings (varimax rotation) of the 10-item perceived-barrier instrument. Bars show the loading of each item on Factor 1 (Structural barriers, navy) and Factor 2 (Psychosocial-normative barriers, dark red). The vertical dotted red lines indicate the ±0.40 threshold for interpretive significance.*

**References cited in supplementary materials**

Brant, R. (1990). Assessing proportionality in the proportional odds model for ordinal logistic regression. Biometrics, 46(4), 1171–1178. doi: 10.2307/2532457

Williams, R. (2016). Understanding and interpreting generalized ordered logit models. Journal of Mathematical Sociology, 40(1), 7–20. doi: 10.1080/0022250X.2015.1112384

Statistics Poland (Główny Urząd Statystyczny). (2022). National Population and Housing Census 2021. Warsaw: GUS.

Statistical Office in Poznań. (2024). Demographic situation of the Greater Poland Voivodeship in 2023. Poznań: Urząd Statystyczny w Poznaniu.
